# Rs488087 single nucleotide polymorphism as predictive risk factor for pancreatic cancers

**DOI:** 10.18632/oncotarget.5627

**Published:** 2015-10-16

**Authors:** Emmanuelle Martinez, Françoise Silvy, Fréderic Fina, Marc Bartoli, Martin Krahn, Fabrice Barlesi, Dominique Figarella-Branger, Juan Iovanna, René Laugier, Mehdi Ouaissi, Dominique Lombardo, Eric Mas

**Affiliations:** ^1^ Aix-Marseille Université, CRO2, Centre de Recherche en Oncologie biologique et Oncopharmacologie, F-13005, Marseille, France; ^2^ INSERM, UMR_S 911, F-13005, Marseille, France; ^3^ LBM- Assistance Publique Hôpitaux de Marseille, Hôpital Nord, Service de Transfert d'Oncologie Biologique, F-13015, Marseille, France; ^4^ Aix-Marseille Université, INSERM, UMR 910, F-13005, Marseille, France; ^5^ Assistance Publique Hôpitaux de Marseille, Hôpital de la Timone-Enfants, Département de Génétique Médicale, F-13005, Marseille, France; ^6^ Assistance Publique Hôpitaux de Marseille, Hôpital Nord, Service d'Oncologie Multidisciplinaire & Innovation Thérapeutique, F-13915, Marseille, France; ^7^ Assistance Publique Hôpitaux de Marseille, Hôpital de la Timone, Service d'Anatomopathologie, F-13005, Marseille, France; ^8^ Aix-Marseille Université, CRCM, Centre de Recherche en Cancérologie de Marseille, F-13009, Marseille, France; ^9^ INSERM, UMR_S 1068, F-13009, Marseille, France; ^10^ CNRS, UMR 7258, F-13009, Marseille, France; ^11^ Assistance Publique Hôpitaux de Marseille, Hôpital de la Timone, Service de Gastroentérologie, F-13005, Marseille, France; ^12^ Assistance Publique Hôpitaux de Marseille, Hôpital de la Timone, Service de Chirurgie Digestive et Viscérale, F-13005, Marseille, France

**Keywords:** SNP, rs488087, pancreatic cancer

## Abstract

Pancreatic cancer (PC) is a devastating disease progressing asymptomatically until death within months after diagnosis. Defining at-risk populations should promote its earlier diagnosis and hence also avoid its development. Considering the known involvement in pancreatic disease of exon 11 of the bile salt-dependent lipase (BSDL) gene that encodes variable number of tandem repeat (VNTR) sequences, we hypothesized upon the existence of a genetic link between predisposition to PC and mutations in VNTR loci. To test this, *BSDL* VNTR were amplified by touchdown-PCR performed on genomic DNA extracted from cancer tissue or blood samples from a French patient cohort and amplicons were Sanger sequenced. A robust method using probes for droplet digital (dd)-PCR was designed to discriminate the C/C major from C/T or T/T minor genotypes. We report that the c.1719C > T transition (SNP rs488087) present in *BSDL* VNTR may be a useful marker for defining a population at risk of developing PC (occurrence: 63.90% in the PC *versus* 27.30% in the control group). The odds ratio of 4.7 for the T allele was larger than those already determined for other SNPs suspected to be predictive of PC. Further studies on tumor pancreatic tissue suggested that a germline T allele may favor Kras G12R/G12D somatic mutations which represent negative prognostic factors associated with reduced survival. We propose that the detection of the T allele in rs488087 SNP should lead to an in-depth follow-up of patients in whom an association with other potential risk factors of pancreatic cancer may be present.

## INTRODUCTION

Pancreatic cancers (PC) represent 10% of all digestive cancers, among which 90% are pancreatic ductal adenocarcinoma (PDAC, www.cancer.org). The survival rate is dramatically low with a case-fatality ratio of about 0.9 with a 5-year survival rate less than 4% in western countries [1]. PDAC could be the second cause of mortality by cancer by the year 2030 [2]. The poor prognosis of this cancer is mainly due to its lack of response to currently available therapies [3, 4] and to unspecific symptoms, the lack of early biological markers, delayed diagnosis and metastasis formation. Risk factors for PC appear to include gender, age, alcohol consumption, smoking and family history of pancreatic cancer. The development and progression of sporadic PC is also associated with altered systemic metabolism occurring in obesity, diabetes and cachexia [5–7]. Recently, several genome-wide association studies identifying PC susceptibility loci (Supplementary Table 1) [8–14]. Although the significance was sometimes high due to large cohorts examined, the predictive value was low as shown by odds ratios. Five hereditary syndromes are associated with an increased risk of PDAC; 1/ The Peutz-Jeghers syndrome caused by germline mutations in the *STK11/LKB1* [15]. 2/ The familial atypical multiple melanoma and mole syndrome caused by mutations in the CDKN2A gene [16]. 3/ Hereditary pancreatitis is also the cause of PDAC with mutation in the cationic trypsinogen gene PRSS1 [17]. 4/ Subjects with *BRCA2* or *PALB2* mutations [18, 19]. 5/ Lastly the Lynch syndrome caused by germline mutations in DNA mismatch repair genes seems linked to a slightly increased risk of developing a pancreatic cancer [20]. *BCRA1* and the ataxia telangiectasia (ATM) gene were also mutated in patients with hereditary PC [21, 22]. Studies have shown that the 8-oxoguanine DNA glycosylase gene (*OGG1*) is a candidate gene for PC development. Links between pancreatic cancer and SNPs in *OGG1* are rather confusing as some SNP are associated with increased [23] or no risk [24] in Japanese population while others seem associated with a protection in Chinese populations [25]. A or B allele carriers in the *ABO* blood group locus on chromosome 9q34 [11] have been linked to PC. Predictive values deduced from those genes were low with modest odds ratios. Thus, a better genetic predictive factor is mandatory for an efficacious follow-up of such an at risk population.

The human bile salt-dependent lipase (BSDL) gene (HGNC: *CEL*) is about 10 kb, with a variable number of tandem repeats (VNTR) encoded by exon 11 [26]. Here we report that SNP rs488087 present in VNTR of BSDL may be predictive of a PC and we designed highly specific probes for droplet digital PCR able to discriminate the c.1719C > T SNP from the wild C/C genotype.

## RESULTS

### c.1719C > T transition (SNP rs488087) in patients with PC

**Figure 1:**
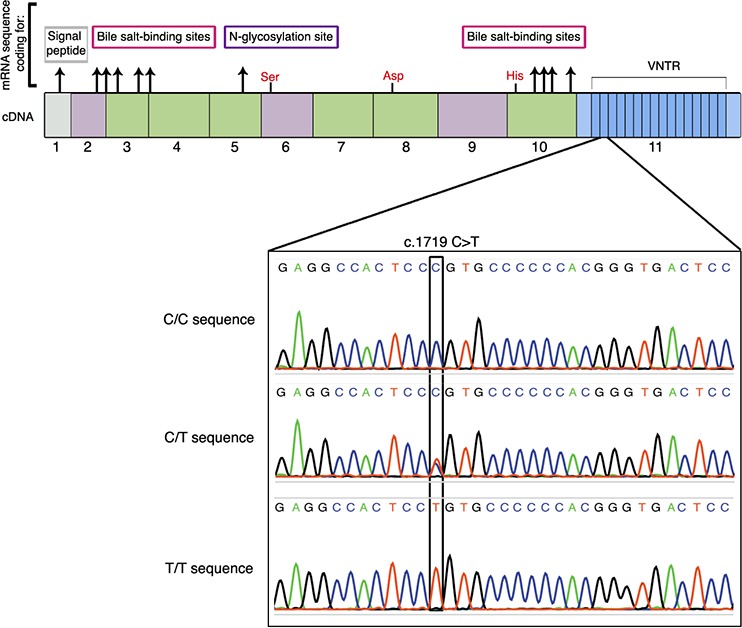
Location of the SNP rs488087 within the cDNA sequence of BSDL Top of the figure shows the 11 exons of the gene with 16 repeated sequences. In red are the three amino acids (Ser-Asp-His) involved in the catalytic site. Electropherograms (lower panel) show the SNP rs488087 in a homozygous sample (C/C), a heterozygous sample (C/T) and a homozygous (T/T) sample.

Detecting genetic risk in the GC-rich VNTR of genes with actual methodologies presents serious limitations [27]. Therefore and in order to search for a genetic predisposition to PC, we sequenced VNTR in *BSDL* in a French cohort of patients with PC and carefully examine electropherograms. Analysis of genomic DNA from tissue extracts highlighted the synonymous SNP rs488087, c.1719C > T, located in the second VNTR sequence of the *BSDL* (Figure 1). When compared to the C/C major genotype and our cancer-free control group, the c.1719C > T transition was associated with PC with *P*-value = 0.0005 and an odds ratio (OR) of 4.72 with a 95% confidence interval (CI) of 1.87 to 11.91 (Table 1). This OR for T allele-holders in the PC population is larger than any previously determined (Supplementary Table 1).

**Table 1:** Association of SNP rs488087 with malignant diseases

Patients (*vs* CTL *n* = 44)	*n*	*X^2^*	*P*-value	Odds Ratio (95 %CI)
PC	36	10.79	0.0005	4.72 (1.87–11.91)
Non-MPD	19	0.58	0.2238	1.56 (0.5–4.9)
OC	78	2.73	0.0492	1.96 (0.88–4.35)

CTL: Control. PC: Pancreatic cancer group. Non-MPD: Non-malignant pancreatic diseases group.

OC: Other cancers group.

The SNP occurrence was 63.90% in patients with sporadic PC (*n* = 36, Table 2), and decreased down to 27.30% in cancer-free control subjects (*n* = 44). The frequency of the T allele was 37.50% (*n* = 72) in the PC cohort *versus* 17.05% (*n* = 88) in the control group (*P*-value = 0.0017). Comparing the T allele frequency in our PC group with the rs488087 SNP data bank (UCSC Genome Browser, *n* = 1275) showed that data remained significant (37.50% versus 23.966%, *P*-value = 0.0041). In addition, the T allele frequency of our control group showed no significant difference with that in the rs488087 data bank (*P*-value = 0.0669). Thus the limited size of our cohorts did not appear to impair the statistical significance of this study. Sequence analyses of patients with non-malignant disease of the pancreas (non-MPD) showed that 7/19 (36.80%) held the T allele (Table 2), showing no significant difference to the control group (*P*-value = 0.224). However, the occurrence of the polymorphism in the non-MPD group was statistically lower than that in the PC group (36.80% *vs* 63.90%, *P*-value = 0.0277). This result obtained on matched non-MPD and PC populations strengthens our conclusion on a link between the c.1719C > T transition and PC.

**Table 2:** Occurrence and allelic frequency of the rs488087 SNP

Occurrence	CTL (*n* = 44)		PC (*n* = 36)		non-MPD (*n* = 19)		OC (*n* = 78)	
	Patient Nb	%	Patient Nb	%	Patient Nb	%	Patient Nb	%
C/C	32	72.70	13	36.10	12	63.20	45	57.70
(C/T) + (T/T)	(9) + (3)	27.30	(19) + (4)	63.90	(6) + (1)	36.80	(29) + (4)	42.30
**Allelic freq.**	**CTL (*n* = 88)**		**PC (*n* = 72)**		**non-MPD (*n* = 38)**		**OC (*n* = 156)**	
	**Allele Nb**	**%**	**Allele Nb**	**%**	**Allele Nb**	**%**	**Allele Nb**	**%**
C	73	82.95	45	62.50	30	78.95	119	76.30
T	15	17.05	27	37.50	8	21.05	37	23.70

freq.: frequency. PC: Pancreatic cancer group. Non-MPD: Non-malignant pancreatic diseases group. OC: Other cancers group.

### Detection of SNP rs488087 by droplet digital PCR (ddPCR)

**Figure 2:**
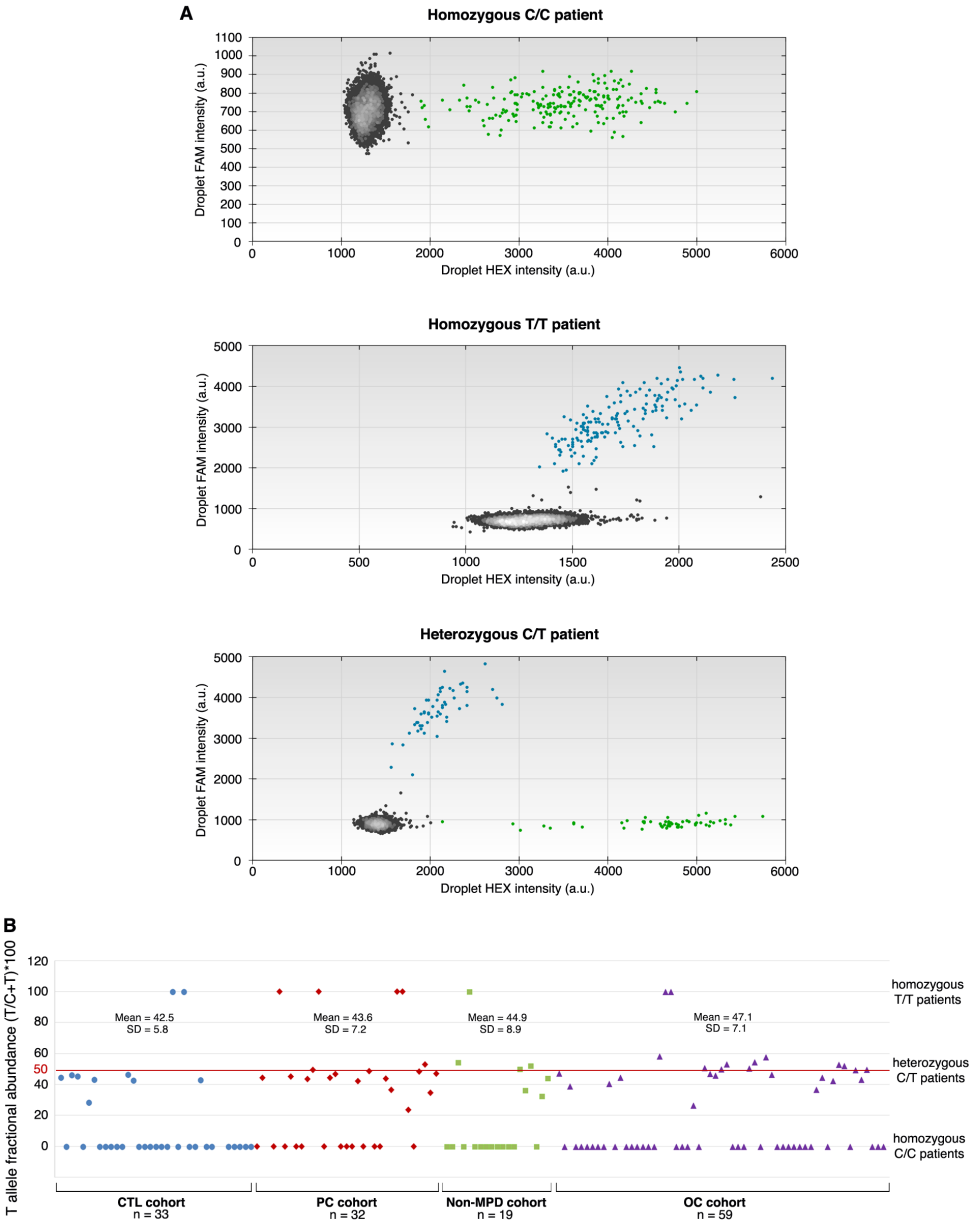
ddPCR analyses The HEX-labeled probe recognizing C target SNP is visualized on the X axis and the 6-FAM labeled probe recognizing T target SNP is visualized on the Y axis. Data from three patients: **A.** Homozygous C patient (upper panel), homozygous T patient (middle panel) and heterozygous C/T patient (lower panel), and **B.** T fractional abundance [(T/T+C)100] for individuals in examined cohorts.

Although it is highly problematical to design specific probes hybridizing in GC-rich domain of any gene and in view of the clinical interest of above given results, we constructed two probes (patented) to discriminate C and T SNP alleles of BSDL by ddPCR. Analyses obtained by ddPCR matched 100% those by Sanger sequencing (*i.e*.143 /143 samples showed C/C alleles or c.1719C > T transition both in ddPCR and in the corresponding Sanger sequencing). This absolute concordance demonstrates the high specificity of these probes to rs488087 SNP and their capacity to allow its simple, rapid and specific detection. Figure 2A shows the three possibilities encountered: homozygous patients C/C (reference sequence), homozygous patients T/T and heterozygous patients C/T. Upon using this method in a cohort of non-pancreatic malignant diseases (other cancers, OC cohort), we found a significant difference between c.1719C > T SNP occurrence among the OC cohort (42.30%) and that in the PC group (63.90%, *P*-value = 0.0161). The SNP occurrence among the OC cohort (42.30%) did not however differ significantly from that of the control group (27.30%), although the *P*-value was border-line (*P*-value = 0.0492) (Table 2). However the SNP occurrence in the whole patient population (OC + non-MPD, *n* = 97) was significantly different (*P*-value = 0.0201) to that determined in the PC cohort. We further have compared PDAC patients to non-PDAC patients associating other PC and OC. In this comparison SNP occurrence still remains statistically significant with a *P*-value = 0.046 (Chi^2^ = 3.96, OR = 3.32, 95% CI = 1.00–5.36). Although significant such *P*-value is border-line, for this reason we have associated the SNP rs488087 to PC and not to PDAC. We also found no significant difference in terms of allelic frequency between OC group (23.70%) and control group (17.05%) (*P*-value = 0.1108), or notably between the OC group and the rs488087 data bank (23.70% *versus* 23.966%, *P*-value = 0.4725). Consequently rs488087 SNP is not associated with non-pancreatic malignant diseases. Furthermore, the SNP occurrence among the OC patient group was not linked to non-MPD (*P*-value = 0.3321).

### Germline character of SNP rs488087 in pancreatic cancer

To determine whether the c.1719C > T transition is of germline or somatic character, we investigated this polymorphism in genomic DNA extracted from whole blood. Results based on sequence analysis of ten patients, show a perfect match between the gDNA obtained from the tumor and that from the whole blood. This correlation was present both in those patients with a normal sequence (7 out of the 10) and in those with a mutated sequence (3 out of the 10 patients, one T/T homozygous and two C/T heterozygous, Supplementary Figure 1). To confirm the germline character of the c.1719C > T transition, we used ddPCR to count the number of copies of DNA target sequence in each of the DNA samples tested by Sanger sequencing. In examined heterozygous samples, the fractional abundance [(T/T+C)*100] was close to 50% (*i.e*. (45.1 +/− 7.2) %, *n* = 52) (Figure 2B) whatever the cohort, thus confirming the germline character [30] of the c.1719C > T transition.

### Relationship between SNP rs488087 and Kras mutations

We have examined *Kras* point mutations as key factors in early tumor progression in our cohort of PDAC patients [29]. Among the 31 PDAC patients, 19 were examined for *Kras* mutations in cancer area of paraffin slides and 14/19 patients (73.7%) were mutated on at least one codon on *Kras*, with 13 patients with *Kras* mutation on codon 12 (Table 3). Two patients (C/T phenotyped) were mutated on exon 3 (D54Y and Q61R) among which one had two Kras hotpoints mutated on exon 2 (G12R) and exon 3 (D54Y). 7/19 (36.8%) of these patients were wild type for SNP of which 6 presented *Kras* point mutation at codon 12, 50% being G12V phenotyped. The remaining patients (12/19, 63.2%) were T allele holders and 7/12 (58.33%) also presented *Kras* mutations. However, examining exon 2 *Kras* mutation subtypes in T allele holders indicated that T allele in rs488087 SNP was mainly associated (6/7 patients, 85.7%) with G12D or G12R Kras phenotype, only one T allele holder patient being G12V (14.3%). Data compilation from the literature [30–34] showed that G12D and G12R mutants together represent 55% of Kras codon 12 mutations (Table 3). Taken all in one these data clearly suggested that holders of the germline T allele on rs488087 SNP further favor somatic mutations on *Kras*, essentially G12D or G12R phenotype.

**Table 3:** Kras mutation subtypes and T-allele holders

Kras mutation subtype	rs488087SNP T allele holders		KRAS mutations *		
	*n* (=7)	%	*n* (=328)	%	Survival range (month) ^Ref : 32,33^
c.35G > A: G12D	4	57.1	185	43.7	8.7–16
c.35G > C: G12R	2	28.6	49	11.6	6.7–18
*Total G12D and G12R*	*6*	85.7	*234*	*55.3*	
c.35G > T: G12V	1	14.3	94	22.2	12.5–16

* References 30–34 were compilated to calculate % of Kras mutation subtype.

## DISCUSSION

The pancreatic BSDL also known as carboxyl-ester lipase (CEL), is secreted into the digestive tract playing a role in cholesterol-esters and lipid-soluble vitamin-esters hydrolysis [35]. Human BSDL gDNA is about 10 kb located on chromosome 9q34.3, with a variable number of tandem repeats (VNTR) in the coding region of exon 11 (from 3 to 21 repeats depending on species and the individual). These VNTR are formed of 33-base pair nearly identical segments [26]. Mutations in VNTR of the BSDL gene cause maturity-onset diabetes of the young (or MODY-8) [36]. Two different single-base deletions, located in repeat 1 and repeat 4, were detected in two families with dominantly inherited diabetes and exocrine dysfunction. Both deletions lead to a frame shift and a premature stop codon, creating a new C-terminal end of the translated BSDL protein and to a misfolded protein responsible for the diabetic pathology [37]. A study performed in a cohort of patients showed the absence of relation between the numbers of VNTR and alcoholic or idiopatic chronic pancreatitis [38] and pancreatic cancer [39]. A recent paper by Fjeld et al. suggested that a crossover between BSDL gene and BSDL pseudogene causes chronic pancreatitis [40]. Finally, Reuss et al. found that the lipase is not differently transcribed in PDAC and normal tissue [41] although VNTR hold oncofetal glyco-epitopes recognized by the 16D10 and J28 monoclonal antibodies which are of potential interest in PDAC therapy [42, 43].

Here we report that SNP rs488087 present in VNTR of BSDL may be predictive of a pancreatic cancer and we designed specific probes for droplet digital PCR to discriminate an at risk population bearing the c.1719C > T SNP from the wild C/C genotype. The odds ratio close to 4.7 for the T allele-holders in the population afflicted with a sporadic PC is significantly larger that those already determined. The fact that the rs488087 SNP cannot distinguish between PDAC and other pancreatic cancers may not be a true matter because more than 90% of sporadic pancreatic cancers are PDAC. A possible bias comes from the size of the cohort in which only 5 patients present non-PDAC pathologies. Many SNP such as SNP rs1042522 in *TP53* (codon 72) has been associated with risk for various human cancers [44]. Therefore we opened this study to lung cancers and to gliomas, two cancers that with pancreatic cancer belong to the stochastic or replicative cluster of cancers [45] and we found that rs488087 may be predictive neither of lung cancers nor of gliomas. Kras somatic mutations were detected in 70–90% of PDAC patients and these mutations which are key factors in early tumor progression [29] mostly affected codon 12 of Kras gene [30–34]. We were able to examine exon 2 *Kras* subtype mutations in cancer tissue and determined that some 86% of patients who were T allele holders were also G12D or G12R. Only one patient over 4 presenting with the G12V phenotype was also T allele holder. Therefore patients with T allele on rs488087 SNP have a tendency to further associate the G12D or G12R, two phenotypes of Kras associated with the worse prognostic and with the lowest patient survival [32, 33]. Although the cancer-free control group allelic frequency perfectly matches with that in the rs488087 data bank and reaches statistical significances when compared to our patient cohorts, data presented here need to be confirmed on larger populations.

In summary, we develop an easy and simple ddPCR that allows us to perfectly discriminate C/C, C/T and T/T allele in a GC rich area of the genome, known to be sources of mis-sequencing [27]. The c.1719C > T transition in rs488087 SNP defines a population at significant risk of developing PC, although further work is necessary to understand the functional consequences of this transition on cell behavior. We propose that the detection of the T allele in rs488087 SNP using our specific probes, should lead to an in-depth follow-up of such patients and an immediate initiation of treatments at the onset of first symptoms of a possible PC to increase efficacy. This follow-up could be particularly pertinent for patients in whom other putative risk factors of developing PC have been found, such as above-described genetic syndromes, diabetes, chronic pancreatitis… It may help also to define a population to be vaccinated once vaccine against PDAC will be available [46, 47].

## MATERIALS AND METHODS

### Cancer-free control (CTL) group

The French control group (Supplementary Table 2) comprised 44 individuals presenting with no cancer-related diseases. Subjects afflicted with infertility were aged between 16 and 54 years (mean = 30.9 years, SD = 8.3) and the male/female ratio was 13/1. Control samples were collected by the Department of Medical Genetics (Timone Hospital, Marseille) between January 2000 and October 2009.

### Pancreatic cancer (PC) group

Tumor tissue samples were taken from 36 patients diagnosed with PC between February 2007 and February 2014 (Gastroenterology and Digestive Surgery departments, Timone Hospital, Marseille, Supplementary Table 3). Patients were aged between 50 and 87 years (mean = 66.8 years, SD = 9.2) and the male/female ratio was 2/3. A definitive diagnosis of pancreatic cancer (adenocarcinoma) was made after histochemical analysis of the resected tissue. The average tumor size was 3.06 cm (SD = 1.27). WHO and TNM stages were determined by a senior pathologist with expertise in the field. One patient had a localized tumor stage 1B, 10 presented a localized tumor stage 2A, 18 had lymph node metastases (stage 2B), 3 were diagnosed at stage 4 (metastatic stage) and 4 patients NC (data not communicated). Survival ranged from 3 to 72 months. According to 2014 hospital records, 22 out of 36 patients with PC died and 9 were still alive at the time of the study. Information is lacking for the remaining patients. The tissue samples were obtained after pancreatic resection (duodeno-pancreatectomy) in patients diagnosed with either adenocarcinoma (PDAC; 30 patients) or non-PDAC pancreatic cancers such as ampulloma, malignant endocrine carcinoma, mucinous cystadenoma (*n* = 6). These patients constituted the pancreatic cancer (PC) group (*n* = 36).

### Non-malignant pancreatic disease (Non-MPD) group

Patients displaying non-malignant pancreatic tissue with chronic calcifying pancreatitis (CCP) or intraductal papillary mucinous tumor (IPMT) constituted the non-malignant pancreatic diseases (non-MPD) group. This cohort collected in the Gastroenterology department (Timone Hospital) comprised 19 patients aged between 40 and 76 years (mean age = 60.9 years, SD = 11.6) (Supplementary Table 4). The male/female ratio was 1.7/1. Four patients presented an IPMT, *i.e*. a cystic tumor of the pancreas with putative pre-neoplastic status, 14 had a CCP, and 1 presented a CCP complicated by a retention cyst. According to the hospital records, all these patients were still alive at the end of 2014.

### Non-pancreatic malignant diseases (other cancers: OC) group

Tissues samples of 78 patients with non-pancreatic malignancies were collected at the Neurobiology, Gastroenterology and Multidisciplinary Oncology & Therapeutic Innovation departments of the Timone and Nord Hospitals (Marseille). Among this cohort, 40 patients were diagnosed with glioblastomas, 2 with sarcomas, 1 with an intestinal cancer with pancreatic metastases, 1 with pancreatobiliary cancer, 20 with lung adenocarcinomas, 9 with epidermoïd lung carcinomas, 1 with undifferentiated carcinoma and 4 with small cell lung carcinomas. Among the 34 lung cancer patients, 3 were non-smokers, 6 remained active smokers and the remaining patients were weaned. Patients (male/female ratio, 1/3) were aged between 20 and 81 years (mean = 60.9 years, SD = 12.3, Supplementary Table 5).

### Generalities on sampling

Material used to detect SNP was either tissue samples (pancreas, glioblastoma, sarcoma, intestinal carcinoma) or blood (lung cancers). All tissue samples were from Caucasian subjects. The protocols for sample collection, sample anonymity, and genomic DNA analysis were approved by the local ethics committee. Written informed consent from all participants was obtained. Samples were stored in the CRO2 biobank (agreement DC 2013–1857) or AP-HM (Assistance Publique - Hôpitaux de Marseille) BioBank (agreement AC-2013–1786). They were kept in a solution of RNA later ^®^ (Life Technologies) from immediately after surgery and subjected to snap-freezing in liquid nitrogen.

### Extraction of genomic DNA (gDNA) from tissue and blood samples

The gDNA was extracted from blood or frozen tissue using the QIAamp mini kit (Qiagen). The extraction is carried out using 600 μL of blood and 25 mg of tissue as recommended. The quantity and quality of extracted gDNA were determined by measuring the absorbance at 260 nm and 280 nm on LVisPlate Omega (BMG Labtech). gDNA samples were stored at −80°C until use.

### Amplifications by TD-PCR

Amplifications were made of sequences encoding BSDL found in the genomic DNA extracted from pancreatic tissue or blood samples using specific primers (Eurogentec). The TD-PCR was performed in a Mastercycler (Eppendorf) in 50 μl (total volume) and comprised 100 ng gDNA or 2 μl of cDNA, 5 μl of 10X enzyme buffer, 10 μl of 5X GCmelt buffer (Ozyme), 200 nM of each primer, 0.4 mM of dNTP mix and 5 U Platinum Taq High fidelity DNA polymerase (Life Technologies). The program was applied as follows: 1 cycle of 5 min at 94°C; 9 cycles of 30 sec at 94°C, 30 sec at 64°C (−1°C per cycle), 1 min at 68°C; 30 cycles of 30 sec at 94°C, 30 sec at 55°C, 1 min at 68°C; 1 cycle of 12 min at 68°C. At the end of PCR, migration on agarose gel was performed to visualize amplified fragments and check sizes.

### Amplicon purification and sequencing

The amplicons obtained were then purified using the DNA extraction kit (Millipore) after electrophoretic migration on agarose gel. Sequencing of the PCR products was carried out according to the Sanger method (Beckman Coulter Genomics, Meylan, France).

### Droplet digital PCR

We used Droplet-Digital PCR (ddPCR) technology that provides a robust quantification of target DNA [28]. Details are described in Supplementary material and methods.

### Detection of kras somatic mutations

*Kras* point mutations were detected on DNA extracted from tumor cell areas from paraffin embedded blade after comparing with the corresponding blade on which the zone containing the tumor cells were delimited by an experienced anatomo-pathologist after coloring with Hematoxyline-Eosine-Safran. Further protocols were given in Supplementary material and methods.

### Statistics

For statistical analysis of results, the Chi^2^ Test was performed using the Graph Pad Prism software (Graph Pad Software).

### Writing assistance

Our manuscript has been edited by Angloscribe, an independent scientific proofreading service provider. Funding source for writing assistance: INSERM.

## SUPPLEMENTARY DATA



## Abbreviations

BSDL: bile-salt-dependent lipase
PDAC: pancreatic ductal adenocarcinoma
PC: pancreatic cancers.
