# Supplementary material for: Rs488087 single nucleotide polymorphism as predictive risk factor for pancreatic cancers
Source: Oncotarget. 2015 Oct 16;6(37):39855–64. doi: 10.18632/oncotarget.5627 (PMC4741865; doi:10.18632/oncotarget.5627)
Supplement: Supplementary file 1 [file oncotarget-06-39855-s001.pdf]

SUPPLEMENTARY DATA

DROPLET DIGITAL PCR

Droplet-Digital PCR (ddPCR) technology provides an absolute count of target DNA copies per input sample without the need for standard curves determination allowing a robust quantification of target DNA [29]. With ddPCR, a sample is partitioned so that individual nucleic acid molecules (15 000 droplets of 1nl, limiting dilution of 0 at 1 DNA copy/droplet) within the sample are localized and concentrated within many separate regions. The partitioning of the sample allows the quantification of molecules according to Poisson's Law. Each part will contain "0" or "1" molecules, or a negative or positive reaction, respectively. After PCR amplification, nucleic acids may be quantified by counting the droplets that contain PCR end-product, positive reactions.

The ddPCR reaction contained: ddPCR supermix for probes (Bio-Rad Laboratories), 100 to 250 mM of each probe (comprising a fluorophore quencher pair), and 450 to 900 mM of each primer and 33 pg to 50 ng of template DNA in a final volume of 20 µL. A total of 22 µL of each reaction mixture was prepared and 20 µl without bubbles were loaded onto a disposable plastic cartridge (Bio-Rad) with 70 µL of droplet generation oil (Bio-Rad) and placed in the QX Droplet Generator (Bio-Rad). The ddPCR began by partitioning the TaqMan reaction mix containing sample DNA into aqueous droplets in oil via the droplet generator, after transfer of droplets to a 96-well PCR plate, followed by a thermocycling protocol (95°C x 10 min; 40 cycles of [94°C x 30s, 54°C x 1 min]; 98°C x 10 min). The PCR plate was then transferred to the QX100 Droplet Reader for automatic reading of samples in each well. Data were analyzed with QuantaSoft analysis software (BioRad Laboratories).

DETECTION OF KRAS SOMATIC MUTATIONS

We extracted DNA from 4 µm formalin fixed paraffin embedded tumor sections blades. Each blade was accompanied by a blade colored with Hematoxyline-Eosine-Safran (HES) on which the zone containing the tumor cells was delimited by an experienced anatomo-pathologist and emphasized by strapping and the proportion of tumor cells indicated. The first stage consisted in dewaxing by Microclearing® (DiaPath, Italy) followed by slow tissue rehydration. DNA were purified by automated extraction on EVO75 (Tecan), according to the Macherey-Nagel protocol; NR (NucleoSpin® 96 Blood; NucleoSpin® 8 Viruses Binding Strips ; Macherey Nagel). We routinely uses qPCR-High Resolution Melting (qPCR-HRM) for *Kras* exon 2, 3 and 4 as a screening technique, allowing to avoid useless sequencing of not mutated samples. The type of mutations defined as “not wild type” by PCR-HRM, is determined by Sanger sequencing (MixBigDye®, Roche) after DNA purification (ExoSap-IT®), pipeting being automated on Evo75®, (Tecan). The sequences are analyzed on 3500 or 3130 Dx Genetic Analyser® (Applied Biosystems). The analytical chain was accredited according to Iso-Norm 15189 (agreement num: 8–1739).

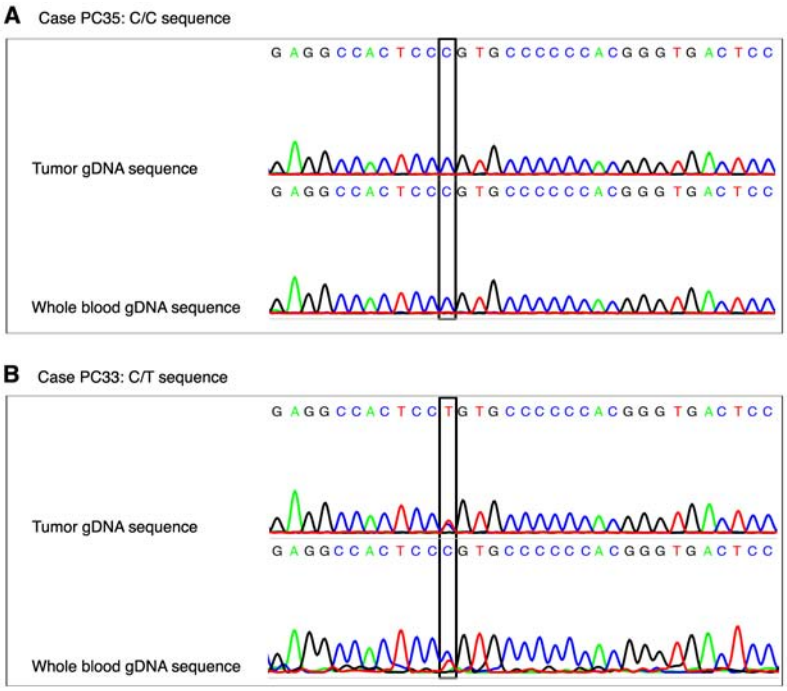

Supplementary Figure S1: Analysis of the electropherogram obtained after the sequencing of the amplicons by Sanger technique on several samples from the same patient. **A.** Case PC35: homozygous C/C patient: concordance between genomic DNA (gDNA) extracted from the tumor and that from whole blood. **B.** Case PC33: heterozygous C/T patient: concordance among gDNA extracted from the tumor and from blood.

Supplementary Table S1: SNPs predictive of a pancreatic cancer

Chromosomes	Locus	Ancestry	SNP Nb	Nearest gene	OR	Ref
1	q32.1	European	37908844	NR5A2	0.77	8, 9
	p36.13	European	16861827	IGSF21	1.7	9
2	p13.3	European	1486134	ETAA1	1.14	10
3	q29	European	9854771	TP63	0.89	10
5	p13.1	European/Chinese	225280	DAB2	0.81/0.74	11,12
	p15.33	European	27336098	CLPTMIL-TERT	0.8/1.19	8
			4011681		1.06	8
6	p25.3	Japanese	9502893	FOXQ1	1.29	13
7	q32.3	European	6971499	LINC-PINT	0.79	11
	q36.2	European	167020	SHH	1.17/1.38	14
		European	172310	SHH	1.17/1.36	14
		European	288746	SHH	1.18/1.39	14
		Japanese	6464375	DPP6	1.13	13
		Japanese	7779540	DPP6	1.14	13
		Japanese	6973850	DPP6	1.1	13
		Japanese	1048768	DPP6	1.13	13
	p13	European	17688601	SUGCT	0.88	10
8	q24.21	European	1561927	PVT1	0.87	11
9	q34	European	505922	ABO locus	1.20/1.44	14
			495828		1.19/1.43	14
			657152		1.19/1.41	14
			630014		0.85/0.71	14
10	q26.11	European/Chinese	12413624	PRLHR	1.23	12
11	p15.4	European	12362504	SBF2	1.4	9
			10500715	SBF2	0.74	9
12	p11.21	Japanese	708224	B1CD1	1.32	13
13	q12.2	European	9581943	PVDX1	1.15	11
	q22.1	European/Chinese	9543325	KFL5	1.26	8,12
			9564966	KFL12	1.21	8
15	q14	European	8028529	none	1.14/1.31	14
			4130461	none	1.10/1.21	14
			4459505	none	1.13/1.28	14
16	q23.1	European	7190458	CTRB1/B2	1.46/0.79	11
17	q25.1	European	11655237	LINC00673	1.26	10
18	p11.21	European	981621	C18orf1	1.39	9
21	q21.3	European/Chinese	372883	BACH1	0.79/0.69	12
	q22.3	European/Chinese	1547374	TFF	0.79/0.79	12
22	q12.1	European	16986825	ZNRF3	1.18	11
	q13.32	European/Chinese	5768709	FAM19A5	0.23	12

Supplementary Table S2: Cancer-free control group

Individuals	Age[1]	Gender[2]	SNP rs 488087
CTL 1	39	0	C/T
CTL 2	31	0	C/C
CTL 3	37	0	C/T
CTL 4	37	1	C/T
CTL 5	39	0	C/C
CTL 6	23	0	C/C
CTL 7	24	0	C/T
CTL 8	38	0	C/C
CTL 9	25	0	C/C
CTL 10	33	0	C/C
CTL 11	34	0	C/C
CTL 12	23	0	C/C
CTL 13	30	0	C/T
CTL 14	34	0	C/T
CTL 15	43	0	C/C
CTL 16	36	0	C/C
CTL 17	34	0	C/C
CTL 18	28	0	C/C
CTL 19	30	1	C/C
CTL 20	47	0	C/C
CTL 21	37	0	T/T
CTL 22	54	0	C/C
CTL 23	41	0	T/T
CTL 24	NC	0	C/C
CTL 25	31	0	C/C
CTL 26	NC	NC	C/T
CTL 27	31	0	C/C
CTL 28	16	0	C/C
CTL 29	31	0	C/T
CTL 30	33	0	C/T
CTL 31	25	0	C/C
CTL 32	33	0	C/C
CTL 33	20	0	C/C
CTL 34	31	0	C/C
CTL 35	21	0	C/C
CTL 36	21	0	T/T

(*Continued*)

Individuals	Age[1]	Gender[2]	SNP rs 488087
CTL 37	36	1	C/C
CTL 38	23	0	C/C
CTL 39	22	0	C/C
CTL 40	29	0	C/C
CTL 41	19	0	C/C
CTL 42	16	0	C/C
CTL 43	40	0	C/C
CTL 44	23	0	C/C

CTL: Control. NC: data not communicated. [1]: in years. [2]: Male 0, Female 1.

Supplementary Table S3: Clinical data of patients with pancreatic cancers (PC)

Patients	Diseases	Age[1]	Gender[2]	Tumor (diameter[3])	TNM[4] T	TNM[4] N	TNM[4] M	Grade[5]	SNP rs488087
PC 1	PDAC	59	0	4.5	2	1	0	2B	C/T
PC 2	PDAC	66	0	3.5	3	0	0	2A	C/T
PC 3	PDAC	54	0	3	3	1	0	4	C/C
PC 4	PDAC	73	1	1.8	3	1	0	2B	C/T
PC 5	PDAC	62	0	2	3	1	0	2B	C/C
PC 6	PDAC	57	1	4.3	4	1	1	4	T/T
PC 7	MC	69	0	NC	NC			NC	C/T
PC 8	PDAC	70	0	2.3	3	1	0	2B	C/C
PC 9	PDAC	63	1	2.5	3	1	0	2B	C/T
PC 10	PDAC	66	0	6	3	0	0	2A	C/C
PC 11	PDAC	58	1	5.2	2	1	0	2B	C/C
PC 12	PDAC	67	1	1.5	3	0	0	2A	C/T
PC 13	PDAC	64	0	4.7	3	1	1	4	C/T
PC 14	PDAC	57	1	6	3	1	0	2B	T/T
PC 15	PDAC	63	1	4.5	3	0	0	2B	C/C
PC 16	PDAC	66	1	2.5	3	0	0	2A	C/T
PC 17	PDAC	66	1	2.5	3	0	0	2A	C/T
PC 18	MA	73	1	2.4	4	1	0	NC	C/C
PC 19	PDAC	79	1	2	3	0	0	2A	C/C
PC 20	PDAC	57	0	3	3	0	0	2A	C/C
PC 21	EC	81	1	3	3	1	0	NC	C/T
PC 22	PDAC	62	0	3	3	0	0	2A	C/C
PC 23	PDAC	53	1	2	1	0	0	2A	C/T
PC 24	EC	67	0	1.3	3	1	0	2B	C/T
PC 25	PDAC	61	1	2.3	3	1	0	2B	C/C
PC 26	MA	60	1	2.5	4	1	0	NC	C/C
PC 27	PDAC	72	0	3	3	1	0	2B	C/T
PC 28	PDAC	69	1	3.5	3	0	0	2A	C/T
PC 29	PDAC	57	1	3.7	3	1	0	2B	T/T
PC 30	PDAC	73	1	2	2	0	0	1B	T/T
PC 31	PDAC	50	1	3	2	1	0	2B	C/T
PC 32	PDAC	87	1	3	3	1	0	2B	C/C

(Continued)

Patients	Diseases	Age[1]	Gender[2]	Tumor	TNM[4]			Grade[5]	SNP rs488087
PC 33	PDAC	85	0	3.3	3	1	0	2B	C/T
PC 34	PDAC	79	1	3.5	3	1	0	2B	C/T
PC 35	PDAC	73	1	4.1	3	1	0	2B	C/T
PC 36	EC	83	0	6.5	NC			2B	C/T

PC: Pancreatic Cancer. PDAC: Pancreatic Ductal Adenocarcinoma. MC: Mucinous Cystadenoma. MA: Malignant Ampulloma.
EC: Endocrine Carcinoma. SNP: single nucleotide polymorphism. NC: data not communicated. [1]: in years. [2]: Male 0, Female 1.
[3]: in centimeters. [4]: TNM stage: T: Tumor, N: Node, M: Metastasis. [5]: Tumor grade (WHO).

Supplementary Table S4: Clinical data of the cohort of patients with non-malignant pancreatic diseases (non-MPD)

Patients	Diseases	Age[1]	Gender[2]	SNP rs488087
non-MPD 1	IMPT	69	0	C/C
non-MPD 2	IMPT	56	0	C/C
non-MPD 3	CCP	61	0	C/T
non-MPD 4	CCP	72	0	C/C
non-MPD 5	CCP	71	1	T/T
non-MPD 6	RT	76	1	C/C
non-MPD 7	CCP	40	1	C/C
non-MPD 8	CCP	73	0	C/C
non-MPD 9	CCP	53	1	C/C
non-MPD 10	IMPT	74	1	C/C
non-MPD 11	IMPT	71	0	C/C
non-MPD 12	CCP	48	0	C/C
non-MPD 13	CCP	53	1	C/C
non-MPD 14	CCP	54	0	C/T
non-MPD 15	CCP	68	0	C/T
non-MPD 16	CCP	45	0	C/T
non-MPD 17	CCP	69	1	C/C
non-MPD 18	CCP	43	0	C/T
non-MPD 19	CCP	62	0	C/T

IMPT: intra-papillary mucinous tumor of the pancreas. CCP: chronic calcifying pancreatitis
RT: Retention cyst. [1]: years. [2]: Male: 0, Female: 1.

Supplementary Table S5: Clinical data of the cohort of patients with non-pancreatic malignancies

Patients	Diseases	Age[1]	Gender[2]	Grade[3]	SNP rs488087
OC 1	GB	78	0	4	C/T
OC 2	ELC	64	0	4	C/C
OC 3	GB	20	0	4	C/T
OC 4	PBC	64	0	2B	C/C
OC 5	GB	59	0	4	C/C
OC 6	LA	37	0	3A	C/C
OC 7	ELC	66	1	3A	C/C
OC 8	LA	79	1	4	C/C
OC 9	GB	52	0	4	C/C
OC 10	GB	51	0	4	C/C
OC 11	GB	63	0	4	T/T
OC 12	GB	76	0	4	C/T
OC 13	LA	77	0	4	C/C
OC 14	LA	74	0	4	C/T
OC 15	GIST	42	1	NC	C/C
OC 16	GB	48	0	4	C/C
OC 17	GB	72	0	4	C/C
OC 18	GB	56	0	4	T/T
OC 19	GB	54	0	4	C/C
OC 20	GB	39	0	4	C/C
OC 21	GB	37	1	4	C/C
OC 22	GB	75	0	4	C/C
OC 23	GB	68	0	4	C/C
OC 24	GB	46	0	4	C/C
OC 25	GB	77	1	4	C/C
OC 26	LA	61	1	4	C/T
OC 27	LA	45	0	4	T/T
OC 28	ELC	74	0	3B	T/T
OC 29	LA	61	0	3B	C/C
OC 30	LA	47	1	4	C/C
OC 31	LA	64	1	4	C/C
OC 32	GB	49	0	4	C/C
OC 33	LA	72	0	4	C/T
OC 34	GB	48	1	4	C/C
OC 35	GB	59	1	4	C/C
OC 36	SCLC	81	0	–	C/T

(Continued)

Patients	Diseases	Age[1]	Gender[2]	Grade[3]	SNP rs488087
OC 37	ELC	63	0	4	C/C
OC 38	LA	73	0	4	C/T
OC 39	LA	48	0	4	C/T
OC 40	SCLC	61	0	-	C/T
OC 41	SCLC	65	0	-	C/C
OC 42	LA	52	0	4	C/C
OC 43	GB	68	0	4	C/T
OC 44	GB	53	0	4	C/C
OC 45	GB	53	0	4	C/T
OC 46	LA	59	0	4	C/C
OC 47	GB	59	1	4	C/C
OC 48	GB	69	0	4	C/C
OC 49	ELC	49	0	2B	C/T
OC 50	GB	73	0	4	C/T
OC 51	GB	63	1	4	C/T
OC 52	GB	70	1	4	C/T
OC 53	LA	79	0	4	C/C
OC 54	ELC	70	0	4	C/C
OC 55	ULC	61	0	4	C/C
OC 56	GB	51	1	4	C/C
OC 57	GIST	34	0	NC	C/C
OC 58	ELC	65	1	4	C/C
OC 59	LA	51	1	4	C/C
OC 60	GB	69	0	4	C/C
OC 61	GB	67	0	4	C/T
OC 62	GB	54	1	4	C/T
OC 63	SCLC	70	0	-	C/T
OC 64	LA	66	0	4	C/C
OC 65	LA	73	0	4	C/T
OC 66	LA	71	0	4	C/T
OC 67	ELC	71	0	4	C/T
OC 68	LA	65	1	4	C/T
OC 69	GB	64	0	4	C/T
OC 70	GB	75	0	4	C/T
OC 71	GB	45	1	4	C/C
OC 72	GB	59	0	4	C/T

(*Continued*)

Patients	Diseases	Age[1]	Gender[2]	Grade[3]	SNP rs488087
OC 73	GB	68	0	4	C/T
OC 74	GB	61	0	4	C/T
OC 75	GB	55	0	4	C/T
OC 76	GB	51	0	4	C/C
OC 77	IC	76	0	1B	C/C
OC 78	ELC	64	0	4	C/C

NC: data not communicated. GB: Glioblastoma. ELC: Epidermoid Lung Carcinoma. LA: Lung Adenocarcinoma. SCLC: Small cell lung carcinoma. GIST: Gastrointestinal Stromal Tumor. PBC: Pancreatobiliary cancer. IC: Intestinal Cancer

ULC: Undifferentiated lung carcinoma. 1: In years. 2: Male 0, Female 1. 3: Tumor grade (WHO).
